# Polymorphic ventricular tachycardia with mutation in KCNJ2: case report

**DOI:** 10.3389/fcvm.2026.1839923

**Published:** 2026-06-16

**Authors:** Cuizhen Zhou, Juan Chen, Cuilan Hou, Tingting Xiao, Li Zhang

**Affiliations:** Department of Cardiology, Shanghai Children’s Hospital, School of Medicine, Shanghai Jiao Tong University, Shanghai, China

**Keywords:** Andersen-Tawil syndrome, case report, KCNJ2, LQTS, polymorphic ventricular tachycardia

## Abstract

Polymorphic ventricular tachycardia (VT), particularly in the absence of structural heart disease, has a strong genetic foundation primarily rooted in mutations affecting cardiac ion channels and associated regulatory proteins. Here, we report two patients with polymorphic VT harboring potassium channel subfamily J member 2 (KCNJ2) gene mutations. The first case is a 13-year-old girl presenting with periodic paralysis, polymorphic VT, and a prolonged QT interval. She was sequentially treated with metoprolol, verapamil, amiodarone, moricizine, and mexiletine, along with three radiofrequency catheter ablation sessions, but the therapeutic effect was unsatisfactory. Genetic testing revealed a *de novo* c.406T > C (p.S136P) variant in the KCNJ2 gene. During a 10-year follow-up period, she never experienced syncope. The second patient is a 5-year-old boy presenting with thumb adduction, polymorphic VT, and a prolonged QT interval. He was treated sequentially with metoprolol and flecainide, and his premature ventricular contraction burden decreased significantly after flecainide therapy. Genetic testing identified a *de novo* c.652C > T (p.A218T) variant in the KCNJ2 gene. This case report updates our understanding of KCNJ2 gene mutations. Arrhythmias due to KCNJ2 mutations respond poorly to antiarrhythmic drugs, but flecainide may be a promising therapeutic option. Arrhythmias associated with KCNJ2 mutations tend to have a more benign clinical course, but identifying mutation carriers at risk of life-threatening arrhythmias remains challenging.

## 1 Introduction

Polymorphic ventricular tachycardia (VT) is a malignant ventricular arrhythmia characterized by variable QRS morphology. It may cause syncope if sustained for several seconds, degenerate into recurrent ventricular fibrillation requiring repeated direct-current cardioversion, or resolve spontaneously. Identifying the underlying etiology of polymorphic VT is critical, as distinct arrhythmic entities with similar electrocardiographic features demand different therapeutic strategies (1). Particularly in the absence of structural heart disease, VT has a strong genetic basis, primarily rooted in mutations affecting cardiac ion channels and associated regulatory proteins. These defects disrupt the delicate balance of ionic currents responsible for the cardiac action potential (AP), leading to arrhythmogenic substrates characterized by abnormal repolarization, early or delayed afterdepolarizations (EADs/DADs), and triggered activity. Multiple genes are associated with VT, including KCNQ1, KCNH2, SCN5A, RYR2, CASQ2, and KCNJ2. Here, we report two patients with polymorphic VT associated with *de novo* pathogenic KCNJ2 missense variants (c.406T > C, p.S136P and c.652C > T, p.A218T), and compare their phenotypes and treatments with a literature review.

## 2 Case presentation

**Figure 1:**
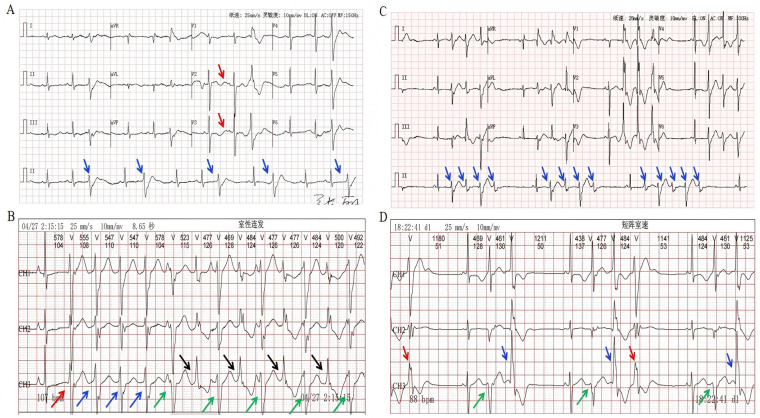
**(A)** resting ECG of case 1 showed sinus rhythm with frequent PVCs (marked with blue arrows). She had QTc of 461 ms and U waves (marked with red arrows). **(B)** Dynamic ECG showed bidirectional and polymorphic ventricular tachycardia of Case 1(different morphologies of ventricular tachycardia were indicated by arrows of different colors). **(C)** Resting ECG of case 2 showed sinus rhythm with ventricular tachycardias (marked with blue arrows). She had QTc of 460 ms. **(D)** Dynamic ECG showed bidirectional and polymorphic ventricular tachycardia of Case 1(different morphologies of ventricular tachycardia were indicated by arrows of different colors).

The first case is a 13-year-old girl who presented with periodic paralysis and arrhythmia for half a year. The electrocardiogram (ECG) showed frequent premature ventricular contractions (PVCs), a prolonged QT interval (the maximum QTc interval = 503 ms) and a prominent U wave in the anterior precordial leads. The dominant PVCs exhibited a right bundle branch block (RBBB) pattern. The 24-hour dynamic electrocardiographic monitor showed 58136 PVCs (PVC burden: 46%) and 1754 episodes of burst VT, manifesting as non-sustained bidirectional ventricular tachycardia (BVT) and polymorphic VT (Figure 1). Her echocardiography was normal (EF = 64%). She was treated with metoprolol, verapamil, amiodarone, moricizine and mexiletine sequentially. Amiodarone had some effect, but the patient developed thyroid dysfunction. She failed to adhere to mexiletine regularly due to gastrointestinal intolerance. Flecainide was not administered due to limited availability in China. She underwent two radiofrequency catheter ablation (RFCA) procedures in our hospital and the third RFCA procedure in other hospital, but the therapeutic effect remained unsatisfactory. During a 10-year follow-up period, she never experienced syncope or cardiac arrest, and her echocardiography remained normal (EF > 60%) despite the persistent high PVC burden. There was no family history of sudden death, syncope, or seizures.

Genetic testing indicated a KCNJ2 mutation, which identified a heterozygous mutation (c.406T > C (p.S136P)). This mutation was absent in both parents, implying a *de novo* mutation, and the c.406T > C (p.S136P) variant has never been reported. Protein stability and pathogenicity were evaluated using three widely used in silico prediction tools: REVEL, SIFT, and MutPred2, with all scores ranging from 0 to 1. Variants with a REVEL score >0.644 indicate a damaging effect; SIFT scores ≤0.05 are considered damaging; MutPred2 scores >0.5 suggest a high probability of pathogenic or functionally deleterious variants. All three tools consistently predicted that the p.S136P variant was damaging and destabilized the protein structure (Table 1).

**Table 1:** Prediction using web-based tools.

Mutation	Web-server	Score	Effect
p.Ser136Phe	REVEL	0.921	Damaging
	SIFT	0.001	Damaging
	MutPred2	0.89	Deleterious
p.Arg218Trp	REVEL	0.982	Damaging
	SIFT	0.000	Damaging
	MutPred2	0.94	Deleterious

**Figure 2:**
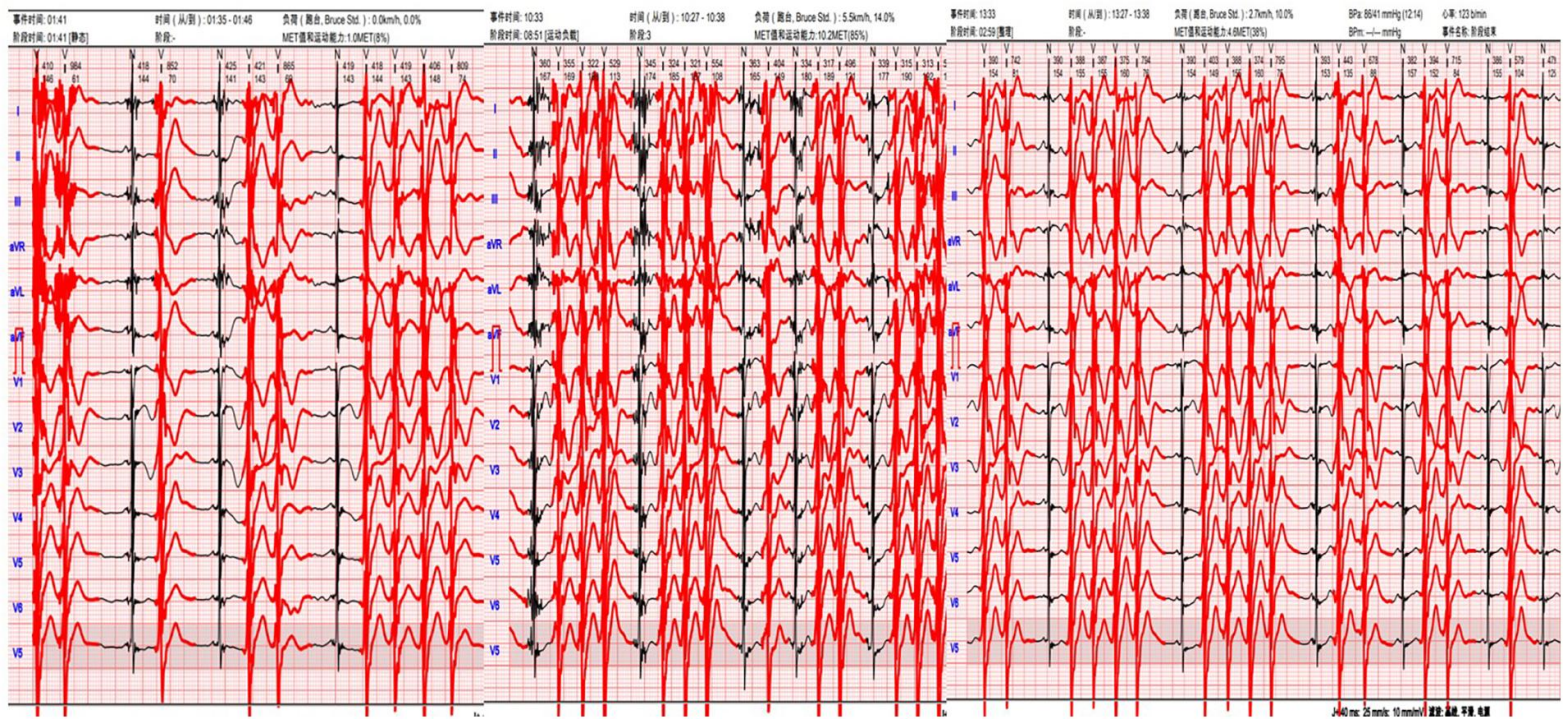
During the exercise and recovery process, frequent short episode of VT occurred, no bidirectional VT or polymorphic VT was observed. No significant changes in premature beats before and after exercise.

The second case is a 5-year-old boy who was asymptomatic but diagnosed with frequent PVCs after a physical examination. He exhibited dysmorphic features such as adduction of thumb. The ECG showed frequent PVCs, a prolonged QT interval (the maximum QTc interval = 490 ms). The dominant PVC also exhibited a right bundle branch block (RBBB) pattern. The 24-hour dynamic electrocardiographic monitor showed 100122 PVCs (PVC burden: 70.2%) and 13866 episodes of VTs (Figure 1), which also manifested with non-sustained BVT and polymorphic VT. His echocardiography (EF = 66%) and cardiac magnetic resonance imaging were normal. A treadmill exercise test was conducted with the Bruce protocol (each stage lasts 3 min). The patient started exercise at the 3rd minute, with a total exercise duration of 8 min and 33 s (Stage 3, 10.2 metabolic equivalents (METs)). The test was terminated due to exhaustion. Frequent short episodes of VT were detected before the start of the exercise and continued during exercise until the heart rate reached 165 bpm, however, no BVT or polymorphic VT was observed (Figure 2). He was treated with metoprolol and flecainide sequentially. Fortunately, his PVC burden decreased to 14.2% after six months of flecainide treatment. There was no familial history of sudden death, syncope, or seizures.

**Figure 3:**
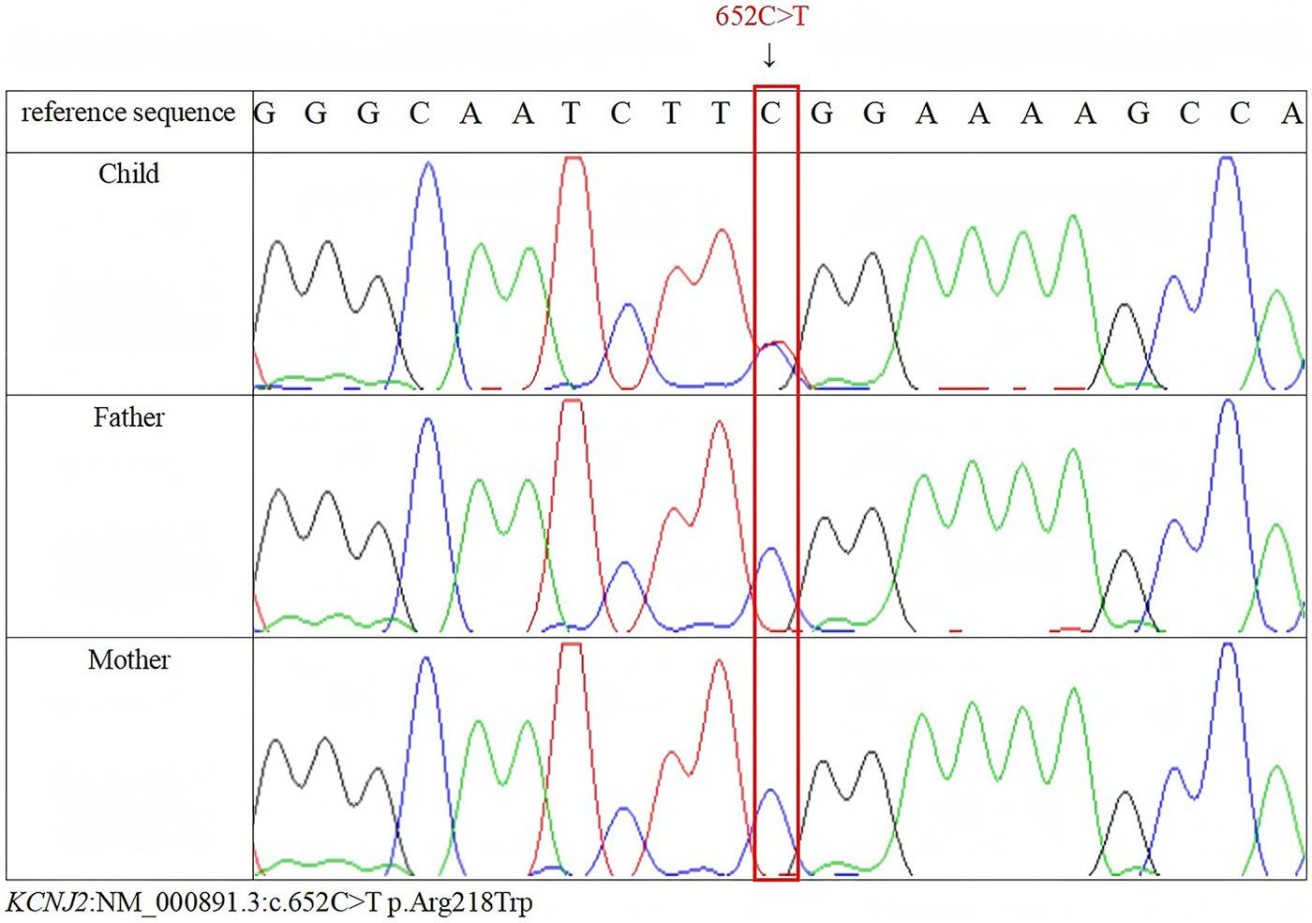
Genetic testing indicates a KCNJ2 mutation, which identified a heterozygous mutation c.652C > T (p.A218T), this mutation was absent in both parents implying a *de novo* mutation.

Genetic testing indicated a KCNJ2 mutation, which identified a heterozygous mutation (c.652C > T (p.A218T)).This mutation was absent in both parents implying a *de novo* mutation (Figure 3). Protein stability and pathogenicity were evaluated using three widely used in silico prediction tools: REVEL, SIFT, and MutPred2, with all scores ranging from 0 to 1. Variants with a REVEL score >0.644 indicate a damaging effect; SIFT scores ≤ 0.05 are considered damaging; MutPred2 scores >0.5 suggest a high probability of pathogenic or functionally deleterious variants. All three tools consistently predicted that the p.A218T variant was damaging and destabilized the protein structure (Table 1).

## 3 Discussion

Polymorphic VT without underlying heart disease demonstrates significant genetic heterogeneity. Primary arrhythmia syndromes include long QT syndrome (LQTS), Brugada syndrome, catecholaminergic polymorphic ventricular tachycardia (CPVT), and short QT syndrome (2). The vast majority of primary cardiac electrical diseases are caused by mutations in genes encoding major ion channel subunits. Congenital LQTS is characterized by prolongation of the QT interval, usually associated with T-wave abnormalities. Torsades de pointes (TdP) can cause syncopal events, ventricular fibrillation and sudden cardiac death (3, 4). Multiple genes are associated with LQTS, including KCNQ1, KCNH2, SCN5A, and KCNJ2. KCNJ2 is a potassium channel-related gene classified as LQT7, also known as Andersen–Tawil syndrome (ATS), which is a rare genetic disorder, characterized by ventricular arrhythmias, periodic paralysis, and physical dysmorphologies (5, 6). KCNJ2 is also a controversial gene in CPVT due to its association with phenotypes that are not representative of CPVT (7, 8). There can be a phenotypic overlap between ATS and CPVT, so careful differentiation is critical. ATS presents with prominent U-wave and marked QTc prolongation on baseline ECG, frequent PVCs with RBBB morphology, and suppression of ventricular arrhythmias at peak exercise. CPVT is associated with PVCs of left bundle branch block (LBBB) morphology and inducible BVT during exercise, whereas resting ECG is usually normal. VT morphology and exercise response help differentiate ATS and CPVT, both of which are characterized by BVT and polymorphic VT (9). In this report, we describe two patients with polymorphic VT caused by KCNJ2 mutations. The first case presented with ventricular tachycardia, a prolonged QT interval, and periodic paralysis, and was diagnosed with ATS based on KCNJ2 gene mutation. The second case manifested with polymorphic VT, a prolonged QT interval, and adduction of thumb; ultimately, ATS was considered based on the KCNJ2 gene mutation and treadmill exercise test results.

The KCNJ2 gene, located on chromosome 17q24.3, encodes the inward rectifier potassium channel protein Kir2.1, a key subunit of inwardly rectifying potassium (Kir) channels. These channels, particularly Kir2.1, exhibit a distinctive biophysical behavior, allowing potassium ions to enter the cell more easily than they exit under specific membrane potentials. In the heart, Kir2.1 is a major contributor to the inward rectifier potassium current (IK1) (10). This current plays a crucial role in repolarization during the final phase of the cardiac action potential and is the primary regulator of the diastolic resting membrane potential in atrial and ventricular cells. To date, only mutations in the KCNJ2 gene have been identified as causes of ATS. Patients with defects in this gene are commonly classified as ATS type 1 (ATS1), while those with unknown mutations are designated as ATS type 2 (ATS2) (11). The first case has a c.406T > C (p.S136P) variant, which is located in exon 2 of the KCNJ2 gene and results in the substitution of serine with proline at position 136 of the encoded Kir2.1 channel protein. The 136th amino acid is situated in the pore helix region of the Kir2.1 channel, adjacent to the selectivity filter of the channel, and is a key conserved site for maintaining the structural stability and potassium ion permeability of the channel. The c.406T > C (p.S136P) variant has not been reported in the literature, though the c.406T > C (p.S136F) variant at the same amino acid position has been identified (10). This mutation prevents the formation of functional homotetrameric channels, resulting in a significant reduction in the inward rectifier potassium current (12). Diminished IK1 depolarizes the resting membrane potential, increasing cellular excitability. It also delays and disperses late repolarization, prolonging action potential duration, which together promotes early afterdepolarizations (EADs) and triggered activity, thereby initiating polymorphic and bidirectional VT (13). The second case has a c.652C > T (p.A218T) variant, causing the substitution of arginine with tryptophan at the 218th amino acid of the Kir2.1 channel protein. This mutation was detected in the proband in a *de novo* form and has been reported in several ATS patients in a heterozygous state (14, 15). The c.652C > T (p.A218G) variant has been confirmed as pathogenic (10, 16, 17). This position is located in the C-terminal phosphatidylinositol 4,5-bisphosphate (PIP2) binding domain of the Kir2.1 channel, a key conserved site for maintaining channel function. C-terminal mutations are more common in typical ATS patients, and arginine at position 218 of the C-terminus is also a relatively common mutation site. The mutation disrupts the binding of the channel to PIP2, resulting in loss of channel function. Functional experiments demonstrated that this variant reduced the binding ability and interaction between Kir2.1 and PIP2, subsequently preventing the formation of functional homomeric complexes (18). Impaired PIP2-dependent gating reduces repolarizing current, prolonging terminal repolarization and enlarging U waves. This increases repolarization heterogeneity, creating a substrate for reentry. It enhances adrenergic-induced EADs, predisposing to bidirectional VT, polymorphic VT, and sudden cardiac arrest (13).

The first patient responded poorly to antiarrhythmic medications. She was administered various medications including metoprolol, verapamil, amiodarone, moricizine, and mexiletine. Fortunately, she has never fainted and her echocardiography is normal so far. β-adrenergic blockers (β-blockers), such as propranolol, are often first-line therapy; however, their efficacy remains controversial (19). In our case, both patients showed no response to β-blockers. Flecainide may serve as an alternative to β-blockers or be used in combination with them. Flecainide acts as a non-selective antagonist of the Nav1.5 cardiac sodium channel, which is encoded by the SCN5A gene and mediates the sodium ion (Na⁺) influx during the upstroke phase of the cardiac action potential. Flecainide has not only been shown to reduce the incidence of arrhythmias in ATS patients during short-term therapeutic interventions but also exhibits efficacy in the long-term management of polymorphic VT when administered over multiple years (20). Georgios A et al. (21) reported that flecainide may suppress ventricular outflow tract PVCs in juveniles, possibly by inhibiting ryanodine receptor-mediated calcium release. The first patient did not receive flecainide because it is unavailable in China, whereas the second patient responded well to flecainide. The exact molecular mechanism of flecainide in ATS is not fully understood. Caballero et al. (22) suggested that the therapeutic effect of flecainide may mainly result from its direct action on the Kir2.1 channel in ATS patients, which can acutely enhance the Kir2.1 channel current. Subsequent studies showed flecainide fails to increase current in R218W-mutant Kir2.1, implying limited efficacy for this variant. Notably, our second patient had a 218-site mutation but responded well to flecainide. The calcium channel inhibitor verapamil has also been used to treat arrhythmias. Verapamil can suppress BVTs, decrease the frequency of polymorphic PVCs and unmask a prolonged QT interval and prominent U waves, but the first patient responded poorly to verapamil. Yang J et al. (23) reported a case of effective arrhythmia control with mexiletine in ATS. As a class Ib antiarrhythmic agent, mexiletine has been proposed as an alternative therapy for LQT2, LQT3, and LQT8 (Timothy syndrome). The mechanism underlying mexiletine-mediated suppression of ventricular arrhythmias in ATS remains incompletely understood. It may involve inhibition of the late sodium current and subsequent modulation of potassium channel function. Another proposed mechanism is that mexiletine shortens ventricular action potential duration (APD) via activation of ATP-sensitive potassium channels (KATP). The most prevalent adverse effects of mexiletine are mild gastrointestinal and neurological symptoms. In the first patient of our cases, mexiletine was discontinued due to intolerable gastrointestinal side effects. As a general rule, avoiding the use of QT prolonging drugs and hypokalemia should be applied in order to prevent life-threatening sustained VTs and sudden death. In patients who show frequent polymorphic VT despite treatment or had previous cardiac arrest, left cardiac sympathetic denervation (LCSD) and implantable cardioverter defibrillator (ICD) can be considered. According to ESC guidelines, ICD implantation is recommended in LQTS patients with previous cardiac arrest, or who experienced syncope while receiving an adequate dose of β-blocker therapy. LCSD should be considered when Beta-blockers are ineffective or contraindicated, ICD therapy is contraindicated or refused, Patients on beta-blockers with an ICD experience multiple shocks (24). Peter et al. (25) confirmed that LCSD significantly reduces syncope, cardiac arrest, and ICD shocks in patients with LQTS and CPVT. However, more evidence is needed to confirm the effectiveness of LCSD in treating VT in patients with ATS. The risk level of polymorphic VT and the prolongation of QT interval in ATS are lower than those of the traditional LQTs and CPVT. Tsuboi Masato et al. (26) confirmed in an ATS model that ATS has a relatively benign phenotype distinct from LQT1 to LQT3. Our first patient had poor responses to antiarrhythmic drugs and radiofrequency ablation treatment. Echocardiography was performed initially because frequent PVCs are associated with cardiomyopathy. Fortunately, she has not developed reduced ejection fraction or PVC-induced cardiomyopathy. She has never had syncope or cardiac arrest, so ICD implantation or LCSD has not been pursued.

The first patient underwent three RFCA procedures, all of which were unsuccessful. Ventricular arrhythmias in ATS are generally thought to originate from the left ventricular Purkinje fibers; however, the mechanism underlying the predominant left ventricular origin of these arrhythmias remains unclear. Previous studies have suggested an association between prominent U-waves and vulnerability to ventricular arrhythmias. Since the structures postulated to contribute to U-wave formation, such as Purkinje fibers and papillary muscles, are more densely distributed in the left ventricle than in the right, it is reasonable to infer that the left ventricle, rather than the right, constitutes the main origin of ventricular arrhythmias in patients with ATS. To date, no published reports exist describing successful RFCA in patients with ATS. Delannoy et al. documented failed RFCA in all five patients in whom the procedure was attempted. While RFCA has previously been pursued in ATS patients with frequent ventricular arrhythmias, successful outcomes have yet to be reported in the literature (27, 28).

Sports-related management is particularly critical, as young individuals are typically highly physically active. Both patients in this report have refrained from competitive sports and strenuous physical activity. At present, the clinical management of athletes with long QT syndrome (LQTS) remains challenging. Whether LQTS patients should be permitted to participate in competitive sports remains controversial among experts. According to ESC guidelines, athletes with a genetically confirmed diagnosis of LQTS should be restricted from competitive sports. In contrast, the American Heart Association (AHA) and American College of Cardiology (ACC) adopt a more lenient approach, allowing participation in competitive sports if the athlete has remained asymptomatic on appropriate therapy for at least 3 months (24, 29, 30).

## 4 Conclusion

When encountering polymorphic VT in children, LQTS and CPVT are more common, with a high risk of syncope and sudden death, as well as a poorer prognosis. ATS is relatively rare and tends to have a more benign outcome; however, identifying KCNJ2 mutation carriers at risk of lethal arrhythmias remains a challenge. By analyzing these two cases, including their gene mutation types and treatment responses, we can further enhance the understanding of this disease. For arrhythmia cases accompanied by neurological symptoms, the possibility of ATS should be considered. Genetic testing is crucial for the diagnosis and treatment of refractory arrhythmic diseases.

## Data Availability

The original contributions presented in the study are included in the article/Supplementary Material, further inquiries can be directed to the corresponding authors.

## References

[1] Viskin S Chorin E Viskin D Hochstadt A Schwartz AL Rosso R. Polymorphic ventricular tachycardia: terminology, mechanism, diagnosis, and emergency therapy. Circulation. (2021) 144:823–39. 10.1161/CIRCULATIONAHA.121.055783 34491774

[2] Wilde A Semsarian C Márquez MF Shamloo AS Ackerman MJ Ashley EA. European Heart Rhythm Association (EHRA)/Heart Rhythm Society (HRS)/Asia Pacific Heart Rhythm Society (APHRS)/Latin American Heart Rhythm Society (LAHRS) expert consensus statement on the state of genetic testing for cardiac diseases. Europace. (2022) 24:1307–67. 10.1093/europace/euac030 35373836 PMC9435643

[3] Priori SG Wilde AA Horie M Cho Y Behr ER Berul C. HRS/EHRA/APHRS expert consensus statement on the diagnosis and management of patients with inherited primary arrhythmia syndromes: document endorsed by HRS, EHRA, and APHRS in May 2013 and by ACCF, AHA, PACES, and AEPC in June 2013. Heart Rhythm. (2013) 10:1932–63. 10.1016/j.hrthm.2013.05.014 24011539

[4] Viskin S. Long QT syndromes and torsade de pointes. Lancet. (1999) 354:1625–33. 10.1016/S0140-6736(99)02107-8 10560690

[5] Crotti L Odening KE Sanguinetti MC. Heritable arrhythmias associated with abnormal function of cardiac potassium channels. Cardiovasc Res. (2020) 116:1542–56. 10.1093/cvr/cvaa068 32227190

[6] Mazzanti A Guz D Trancuccio A Pagan E Kukavica D Chargeishvili T. Natural history and risk stratification in Andersen-Tawil syndrome type 1. J Am Coll Cardiol. (2020) 75:1772–84. 10.1016/j.jacc.2020.02.033 32299589

[7] Leenhardt A Lucet V Denjoy I Grau F Ngoc DD Coumel P. Catecholaminergic polymorphic ventricular tachycardia in children. A 7-year follow-up of 21 patients. Circulation. (1995) 91:1512–9. 10.1161/01.cir.91.5.1512 7867192

[8] Walsh R Adler A Amin AS Abiusi E Care M Bikker H. Evaluation of gene validity for CPVT and short QT syndrome in sudden arrhythmic death. Eur Heart J. (2022) 43:1500–10. 10.1093/eurheartj/ehab687 34557911 PMC9009401

[9] Inoue YY Aiba T Kawata H Sakaguchi T Mitsuma W Morita H. Different responses to exercise between Andersen-Tawil syndrome and catecholaminergic polymorphic ventricular tachycardia. Europace. (2018) 20:1675–82. 10.1093/europace/eux351 29309601

[10] Plaster NM Tawil R Tristani-Firouzi M Canún S Bendahhou S Tsunoda A. Mutations in Kir2.1 cause the developmental and episodic electrical phenotypes of Andersen’s syndrome. Cell. (2001) 105:511–9. 10.1016/s0092-8674(01)00342-7 11371347

[11] Donaldson MR Yoon G Fu YH Ptacek LJ. Andersen-Tawil syndrome: a model of clinical variability, pleiotropy, and genetic heterogeneity. Ann Med. (2004) 36 Suppl 1:92–7. 10.1080/17431380410032490 15176430

[12] Zhang L Benson DW Tristani-Firouzi M Ptacek LJ Tawil R Schwartz PJ. Electrocardiographic features in Andersen-Tawil syndrome patients with KCNJ2 mutations: characteristic T-U-wave patterns predict the KCNJ2 genotype. Circulation. (2005) 111:2720–6. 10.1161/CIRCULATIONAHA.104.472498 15911703

[13] Moreno-Manuel AI Gutiérrez LK Vera-Pedrosa ML Cruz FM Bermúdez-Jiménez FJ Martínez-Carrascoso I. Molecular stratification of arrhythmogenic mechanisms in the Andersen Tawil syndrome. Cardiovasc Res. (2023 May 2) 119(4):919–32. 10.1093/cvr/cvac118 35892314 PMC10153646

[14] Tristani-Firouzi M Jensen JL Donaldson MR Sansone V Meola G Hahn A. Functional and clinical characterization of KCNJ2 mutations associated with LQT7 (Andersen syndrome). J Clin Invest. (2002) 110:381–8. 10.1172/JCI15183 12163457 PMC151085

[15] Luo S Xu M Sun J Qiao K Song J Cai S. Identification of gene mutations in patients with primary periodic paralysis using targeted next-generation sequencing. BMC Neurol. (2019) 19:92. 10.1186/s12883-019-1322-6 31068157 PMC6505267

[16] Bendahhou S Donaldson MR Plaster NM Tristani-Firouzi M Fu YH Ptácek LJ. Defective potassium channel Kir2.1 trafficking underlies Andersen-Tawil syndrome. J Biol Chem. (2003) 278:51779–85. 10.1074/jbc.M310278200 14522976

[17] Wang Q Zhao Z Shen H Bing Q Li N Hu J. The clinical and genetic heterogeneity analysis of five families with primary periodic paralysis. Channels (Austin). (2021) 15:20–30. 10.1080/19336950.2020.1857980 33345742 PMC7757828

[18] Cruz FM Macías Á Moreno-Manuel AI Gutiérrez LK Vera-Pedrosa ML Martínez-Carrascoso I. Extracellular Kir2.1(C122Y) mutant upsets Kir2.1-PIP(2) bonds and is arrhythmogenic in Andersen-Tawil syndrome. Circ Res. (2024) 134:e52–52e71. 10.1161/CIRCRESAHA.123.323895 38497220 PMC11009053

[19] Bökenkamp R Wilde AA Schalij MJ Blom NA. Flecainide for recurrent malignant ventricular arrhythmias in two siblings with Andersen-Tawil syndrome. Heart Rhythm. (2007) 4:508–11. 10.1016/j.hrthm.2006.12.031 17399642

[20] Fox DJ Klein GJ Hahn A Skanes AC Gula LJ RK Y. Reduction of complex ventricular ectopy and improvement in exercise capacity with flecainide therapy in Andersen-Tawil syndrome. Europace. (2008) 10:1006–8. 10.1093/europace/eun180 18621769

[21] Christou GA Letsas KP Konstandi M Christou MA Christou KA Kyriakopoulos C. Case report: high efficacy of low-dose flecainide as an add-on therapy to a beta-blocker for treating a high burden of idiopathic ventricular arrhythmias in a juvenile athlete. Front Cardiovasc Med. (2025) 12:1537078. 10.3389/fcvm.2025.1537078 40384969 PMC12081362

[22] Caballero R Dolz-Gaitón P Gómez R Amorós I Barana A González de la Fuente M. Flecainide increases Kir2.1 currents by interacting with cysteine 311, decreasing the polyamine-induced rectification. Proc Natl Acad Sci U S A. (2010) 107:15631–6. 10.1073/pnas.1004021107 20713726 PMC2932566

[23] Yang J Li K Lv T Xie Y Liu F Zhang P. Case report: mexiletine suppresses ventricular arrhythmias in Andersen-Tawil syndrome. Front Cardiovasc Med. (2022) 9:992185. 10.3389/fcvm.2022.992185 36093155 PMC9453449

[24] Priori SG Blomström-Lundqvist C Mazzanti A Blom N Borggrefe M Camm J. 2015 ESC guidelines for the management of patients with ventricular arrhythmias and the prevention of sudden cardiac death: the task force for the management of patients with ventricular arrhythmias and the prevention of sudden cardiac death of the European Society of Cardiology (ESC). Endorsed by: Association for European Paediatric and Congenital Cardiology (AEPC). Eur Heart J. (2015) 36:2793–867. 10.1093/eurheartj/ehv316 26320108

[25] Schwartz PJ Ackerman MJ. Cardiac sympathetic denervation in the prevention of genetically mediated life-threatening ventricular arrhythmias. Eur Heart J. (2022) 43:2096–102. 10.1093/eurheartj/ehac134 35301528 PMC9459868

[26] Tsuboi M Antzelevitch C. Cellular basis for electrocardiographic and arrhythmic manifestations of Andersen-Tawil syndrome (LQT7). Heart Rhythm. (2006) 3:328–35. 10.1016/j.hrthm.2005.11.026 16500306 PMC1474110

[27] Keegan R Onetto L Gregorietti F Urruti R Di Biase L. Catheter ablation of frequent monomorphic ventricular arrhythmias in Andersen-Tawil syndrome: case report and focused literature review. J Interv Card Electrophysiol. (2023) 66:729–36. 10.1007/s10840-021-01077-w 34665385

[28] Delannoy E Sacher F Maury P Mabo P Mansourati J Magnin I. Cardiac characteristics and long-term outcome in Andersen-Tawil syndrome patients related to KCNJ2 mutation. Europace. (2013) 15:1805–11. 10.1093/europace/eut160 23867365

[29] Christou GA Vlahos AP Christou KA Mantzoukas S Drougias CA Christodoulou DK. Prolonged QT interval in athletes: distinguishing between pathology and physiology. Cardiology. (2022) 147:578–86. 10.1159/000526385 35947943

[30] Priori SG Wilde AA Horie M Cho Y Behr ER Berul C. Executive summary: HRS/EHRA/APHRS expert consensus statement on the diagnosis and management of patients with inherited primary arrhythmia syndromes. Europace. (2013) 15:1389–406. 10.1093/europace/eut272 23994779

